# Yeast Beta-Glucan Supplementation with Multivitamins Attenuates Cognitive Impairments in Individuals with Myalgic Encephalomyelitis/Chronic Fatigue Syndrome: A Randomized, Double-Blind, Placebo-Controlled Trial

**DOI:** 10.3390/nu15214504

**Published:** 2023-10-24

**Authors:** Marcos Lacasa, Jose Alegre-Martin, Ramon Sanmartin Sentañes, Luisa Varela-Sende, Joanna Jurek, Jesus Castro-Marrero

**Affiliations:** 1 E-Health Center, Universitat Oberta de Catalunya, 08018 Barcelona, Spain; mlacasaca@uoc.edu; 2 Myalgic Encephalomyelitis/Chronic Fatigue Syndrome Research Unit, Division of Rheumatology, Vall d´Hebron University Hospital Research Institute, Universitat Autònoma de Barcelona, 08035 Barcelona, Spain; jose.alegre@vallhebron.cat (J.A.-M.); ramon.sanmartin@vallhebron.cat (R.S.S.); joanna.michalina.jurek@gmail.com (J.J.); 3 Clinical Research Department, VITAE Health Innovation, Montmeló, 08160 Barcelona, Spain; lvarela@vitae.es

**Keywords:** chronic fatigue syndrome, beta-glucan, zinc, vitamin D_3_, vitamin B_6_, myalgic encephalomyelitis, mitochondria, non-restorative sleep, quality of life

## Abstract

This research aimed to examine the potential alleviative effects of beta-glucan administration on fatigue, unrefreshing sleep, anxiety/depression symptoms and health-related quality of life in ME/CFS. A 36-week unicenter, randomized, double-blind, placebo-controlled trial was conducted in 65 ME/CFS patients, who were randomly allocated to one of two arms to receive four capsules each one of 250 mg beta-glucan, 3.75 µg vitamin D3, 1.05 mg vitamin B6, and 7.5 mg zinc (n = 35), or matching placebo including only microcrystalline cellulose as an excipient (n = 30) once daily. The findings showed that the beta-glucan supplementation significantly improved cognitive fatigue (assessed with FIS-40 scores) after the 36-week treatment compared to the baseline (*p* = 0.0338). Taken together, this study presents the novel finding that yeast-derived beta-glucan may alleviate cognitive fatigue symptoms in ME/CFS. Thus, it offers valuable scientific insights into the potential use of yeast beta-glucan as a nutritional supplement and/or functional food to prevent or reduce cognitive dysfunction in patients with ME/CFS. Further interventions are warranted to validate these findings and also to delve deeper into the possible immunometabolic pathomechanisms of beta-glucans in ME/CFS.

## 1. Introduction

Myalgic encephalomyelitis or chronic fatigue syndrome (ME/CFS) is a complex debilitating disorder affecting approximately 17 million people worldwide with a prevalence ranging between 0.2 and 2.6% [1] and a higher frequency in women [2]. ME/CFS is characterized by unexplained and persistent post-exertional fatigue lasting for at least six months, which worsens with physical or mental activity and is not relieved by rest or sleep [3]. ME/CFS is often accompanied by a cluster of other symptoms such as cognitive impairments, impaired immune function, neuroendocrine alterations, and autonomic dysfunction [4]. The condition represents a considerable public health problem and is a cause of severe disability in society, with a marked impact on professional activities and on social and personal relationships [5,6]. All these symptoms can significantly compromise the quality of life of sufferers, as they limit the ability of ME/CFS patients to perform daily tasks [7,8].

In addition, the sleep disturbances commonly reported in ME/CFS patients have been shown to exaggerate these cognitive symptoms and make them more difficult to control [9,10]. At present, there are no specific tests for diagnosing ME/CFS, and so a comprehensive clinical assessment is essential, especially when evaluating the impact of fatigue, quality of life, anxiety/depression symptoms, and health-related quality of life; this assessment should include consultation of medical records, physical examination, laboratory testing, and neuroimaging techniques, along with validated neuropsychiatric tools [11,12]. Although the exact pathophysiological mechanisms underlying cognitive dysfunction are not yet fully understood, alterations in the autonomic nervous system and cerebral blood flow have been proposed as possible causes [13]. In addition, poor sleep quality in ME/CFS has been shown to worsen mental and muscular fatigue, causing intolerance to physical exercise and presenting clinically as myalgia and insufficient/poor blood flow to the brain with additional disturbances in energy production [10].

Functional alterations of redox metabolism, gene expression, and mitochondrial biomarkers in peripheral blood mononuclear cells (PBMC), aerobic oxidative metabolism, decreased aerobic activity, and deconditioning after physical exercise have also been described in ME/CFS [4,14]. High levels of pro-inflammatory cytokines, such as interleukin-1 (IL-1), tumor necrosis factor (TNF-α), and elastase, as well as high oxidative and nitrosative stress, may have multiple effects; they can inhibit mitochondrial respiration, decrease electron transport chain activity and change mitochondrial membrane potential, increase the mitochondrial membrane permeability, and interfere with the ATP production, finally leading to mitochondrial inactivation [15]. Along with morning fatigue, exercise intolerance, and alterations in concentration and memory, sleep disturbances is a particular salient feature of ME/CFS, in the form of unrefreshing sleep, insomnia, hypersomnia, presence of nightmares and/or bad dreams, restless leg syndrome, and sleep apnea syndrome [16].

In addition, many individuals with ME/CFS report gastrointestinal complaints such as irritable bowel syndrome (IBS), a common functional disorder of the gastrointestinal tract, characterized by abdominal pain or discomfort and altered bowel habit. Although the exact mechanism of the gut disturbances in ME/CFS needs to be explored further, the high frequency of IBS diagnoses in ME/CFS patients suggests that impaired composition of intestinal microbiota along with immune dysfunction and increased inflammation beyond the gut may play a role in the onset and development of the illness [17]. The involvement of a bidirectional link between the gut and the central nervous system (CNS) in ME/CFS, known as the gut–brain axis, was supported by studies showing improvements of various symptoms, including anxiety/depression, sleep quality, and neurocognitive impairments after probiotic supplementation interventions [18,19]. Moreover, the dysbiosis of the gut microbiome observed in ME/CFS was linked to the elevated levels of circulating inflammatory mediators, possibly due to an increase in intestinal permeability that allowed for bacterial translocation [20].

Studies investigating the role of short-chain fatty acids (SCFA) in ME/CFS pathogenesis have suggested that SCFA deficiency, in particular butyrate-producing bacteria, is associated with fatigue severity in these individuals [21], as well in other chronic comorbidity conditions, including IBS [22], cancer [23], multiple sclerosis [24], and type 1 diabetes [25]. In view of these early findings, enhancing butyrate production in the gut may have a beneficial therapeutic effect in ME/CFS. One way to increase SCFA levels is through the supplementation of prebiotics, in form of non-digestible polysaccharides (NPS), which upon fermentation, can selectively stimulate the growth and/or activity of anti-inflammatory bacteria in the gut [25]. Beta-glucan, an example of an NPS found as glucose polymers in yeast, fungi, algae, and cereals, such as oats and barley, can promote the growth of probiotic bacteria and increase the production of SCFA, in particularly butyrate [26,27].

Although many studies have investigated the health benefits of beta-glucan, including the prevention and treatment of chronic gut inflammation and conditions related to metabolic disturbances and neurodegeneration, its use in ME/CFS is still under investigation [26,28,29]. Experimental pre-clinical studies using murine models have shown that beta-glucan supplementation can reduce microbial translocation, restore immune homeostasis, and decrease fatigue [30]; these early findings are now under investigation in clinical trials (https://clinicaltrials.gov, accessed on 11 May 2023; NCT05726435 and NCT05524688). The accompanying synergistic improvements in terms of blood pressure, decreased confusion, and improved mood can also help to reduce perceived fatigue [30,31].

Furthermore, by promoting the activity of the desired intestinal microbiota and limiting the growth of pathogens, beta-glucan can play an important role in maintaining proper gut function and thus prevent chronic inflammation. For example, the use of a yeast-derived beta-glucan was effective in reducing hyperpermeability in ileal specimens from patients with Crohn’s disease [32], and it also reduced chronic abdominal pain associated with altered bowel habits in an experimental IBS murine model [33]. Similar improvements in bloating, flatulence, and abdominal pain were also observed in a clinical intervention with a mixture of beta-glucan, inositol, and digestive enzymes in IBS patients [34]. This finding may be of particular importance in ME/CFS, as the gastrointestinal issues frequently reported by these patients are often associated with psychological distress and increased release of corticotrophin-releasing hormone, which is known to be involved in the stimulation the hypothalamic–pituitary–adrenal (HPA) axis and is associated with major depression and IBS [35,36].

Thus, beta-glucan supplementation is associated with numerous health benefits including improved gut health, enhanced immune function, and reduced fatigue, and may provide a potential therapeutic advantage for ME/CFS. The present randomized placebo-controlled trial aimed to assess the effect of ImmunoVita^®^, a dietary supplement composed of yeast-derived beta-glucan combined with vitamin D3, vitamin B6, and zinc on fatigue, sleep problems, anxiety/depression, and health-related quality of life in ME/CFS.

## 2. Materials and Methods

### 2.1. Participants

**Figure 1:**
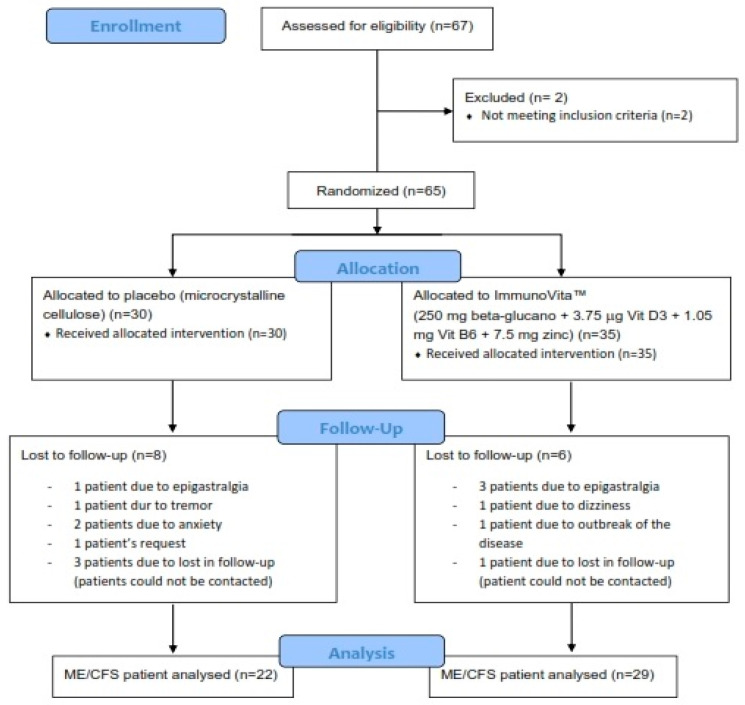
Consolidated Standards of Reporting Trials (CONSORT) flow diagram illustrating the steps of screening, enrollment, assignment, and follow-up of the study participants.

This study was conducted in 67 Caucasian ME/CFS patients consecutively recruited from a single outpatient tertiary referral center (ME/CFS Clinical Unit, Vall d’Hebron University Hospital, Barcelona, Spain) from September 2021 to December 2022. Figure 1 shows a flowchart of the participants prior to analysis. Patients were potentially eligible for the study if they were female, aged 18 years or older, and had a confirmed diagnosis of ME/CFS according to the 1994 CDC/Fukuda case definition [37].

Exclusion criteria comprised participation in another intervention within 30 days prior to study inclusion; inability (in the opinion of the investigator) to follow the instructions or to complete the treatment satisfactorily; failure to provide signed informed consent; consumption of certain drugs/supplements that might influence outcome measures in the last 90 days or whose withdrawal might be a relevant problem, anticoagulant treatment, pregnancy or breast-feeding, smoking, alcohol intake or substance abuse, BMI ≥ 25 kg/m^2^, and hypersensitivity to any of the components of treatments. Patients with missing data from the follow-up visits were considered to have dropped out. The participants signed an informed consent form prior to the study.

### 2.2. Intervention Protocol

Of the 67 eligible ME/CFS participants screened, two were excluded. The remaining 65 participants were allocated to treatment by an independent investigator not otherwise involved in the intervention, using a list of random numbers generated by a computer program. The participants were randomly assigned in a double-blind fashion in a 1:1 ratio to receive either active treatment (n = 35) or a matching placebo (n = 30) in the form of four capsules daily for nine months. They were instructed to ingest the capsules on an empty stomach, 30 minutes before breakfast and dinner, only with water. The intervention comprised the intake of a food supplement containing beta-glucan, vitamin D3, vitamin B6, and zinc. The placebo group received the same food supplement containing only microcrystalline cellulose instead of active ingredients.

Safety information recorded at all study visits included data on adverse events, vital signs, and the results of the general physical examination. Adverse events, including serious ones, were reviewed throughout the trial by the independent medical monitor, the steering committee, and the independent data and safety monitoring board. Provision was made for investigator-initiated temporary or permanent dose reductions or suspensions due to adverse effects.

During the study, nine subjects dropped out due to adverse events, specifically five in the intervention group (three epigastralgias, one dizziness, and one outbreak of the disease) and four in the placebo group (one epigastralgia, one tremor, and two anxiety episodes). Four patients were lost to follow-up (one in the intervention group and three in the placebo group). Finally, one patient in the placebo group was withdrawn at her own request. The remaining 51 cases of ME/CFS (78%, 22 in the placebo group and 29 in the intervention group) completed all the study protocol procedures and were included in the overall analysis of outcome measures as displayed in Figure 1.

### 2.3. Testing of Dietary Supplements

Patients randomized to the intervention’s experimental group received a daily dose of four capsules composed of 250 mg beta-glucan, 3.75 µg vitamin D3, 1.05 mg vitamin B6, and 7.5 mg zinc, plus microcrystalline cellulose and plant capsule as excipients. The placebo composition was the same capsule with microcrystalline cellulose without any active ingredients. The treatments were identical in terms of size, color, opacity, shape, presentation, and packaging. All capsules were manufactured and donated by Vitae Health Innovation S.L. (Montmeló, Barcelona, Spain). The study pharmacist recorded all treatments supplied on the medication-dispensing forms along with their original prescription.

### 2.4. Study Design and Procedures

**Figure 2:**
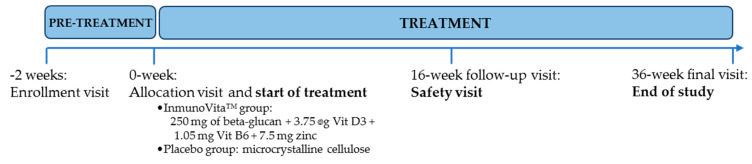
Summary of the study schedule at each visit during the clinical trial.

The trial was a 36-week long, single-center, randomized, double-blind, placebo-controlled study. Clinical visits and trial design of both groups are detailed in Figure 2. After an oral explanation of the study, all participants provided their written consent prior to the commencement of the study, and they received no compensation for their participation.

Patients were evaluated at baseline, at a 16-week follow-up (safety) visit, and then at a 36-week visit by the site investigator. Changes in symptoms were assessed through validated self-report questionnaires completed by participants under the supervision of two trained investigators (J.C.-M. and J.A.). Compliance was checked through medication logs. The use of concomitant medications was tracked at the 36-week visit. The study protocol was reviewed and approved by the local IRB at the participating site (Clinical Research Ethics Committee, Vall d’Hebron University Hospital, Barcelona, Spain, under protocol reference InmunoVitaME-PR(AG)-447-2019, approved on 28 February 2019).

The study protocol was conducted in accordance with the guidelines of the Declaration of Helsinki, the current Spanish regulations on clinical research, and the standards of good clinical practice of the European Union. It also followed the Consolidated Standards of Reporting Trials (CONSORT) guidelines. The current clinical trial was registered on https://clinicaltrials.gov as NCT04301609.

### 2.5. Primary Endpoint

#### Fatigue Perception

The primary endpoint was the change in self-reported fatigue perception assessed by using the validated Fatigue Impact Scale (FIS-40) questionnaire from the baseline to the final (week 36) study visit. Briefly, the FIS-40 comprises 40 items divided into three domains that describe how perceived fatigue impacts on cognitive (10 items), physical (10 items), and psychosocial functioning (20 items) over the previous four weeks. Each item is scored from 0 (no fatigue) to 4 (severe fatigue). The total score is calculated by adding together responses from the 40 questions (score range 0–160). Higher scores indicate more functional limitations due to severe fatigue [38].

### 2.6. Secondary Endpoints

The secondary outcome measures included changes in sleep disturbance, anxiety/depression symptoms, and health-related quality of life (HRQoL) on the validated self-reported questionnaires.

#### 2.6.1. Sleep Quality

Sleep quality was assessed using the self-administered 19-item Pittsburgh Sleep Quality Index (PSQI) questionnaire. Scores are obtained on each of seven domains of sleep quality: subjective sleep quality, sleep latency, sleep duration, habitual sleep efficiency, sleep perturbations, use of sleeping medication, and daytime dysfunction. Each component is scored from 0 to 3 (0 = no sleep problems and 3 = severe sleep problems). The global PSQI score ranges from 0 to 21 points, with scores of >5 indicating poorer sleep quality [39].

#### 2.6.2. Anxiety and Depression

Severity of anxiety/depression symptoms was assessed using the Hospital Anxiety and Depression Scale (HADS), a validated self-reported tool composed of 14 items (seven related to anxiety symptoms and seven to depression). Each item on the HADS questionnaire is scored from 0–3, and so scores range from 0 to 21; scores of 0–7 are interpreted as normal, 8–10 as mild, 11–14 as moderate, and 15–21 as severe for either anxiety or depression. The total HADS score ranges from 0 (no anxiety or depression) to 42 (severe anxiety and depression) [40].

#### 2.6.3. Health-Related Quality of Life

The 36-item Short Form Health Survey (SF-36) was used to assess HRQoL. The SF-36 is a broadly based self-reported survey of health-related physical and mental functioning statuses. It assesses functioning on eight subscales including domains of physical functioning, physical role, bodily pain, general health, social functioning, vitality, emotional role, and mental health, and two general subscales covering the physical and mental health domains rated on a scale from 0–100. Lower scores indicate a more negative impact on health and daily functioning [41].

### 2.7. Sample Size Estimation and Power Analysis

This trial was the first exploratory, population-based proof-of-concept study in people with ME/CFS. Sixty participants were enrolled with 30 patients being randomly allocated to each arm.

### 2.8. Compliance Monitoring and Adverse Events

All participants were asked to return any remaining study products after the intervention. Adherence was measured by calculating all remaining capsules for all patients, including withdrawals. Participants who did not take the supplement for more than two days (either consecutive or non-consecutive) were considered non-compliant (n = 0). All adverse events following administration and intake of the study product were monitored until the end of the study.

### 2.9. Statistical Analysis

A total of 51 patients were analyzed, divided into two groups: active (n = 29) and placebo (n = 22). The variables were evaluated (1) at different times (questionnaire domains) and (2) at the time of inclusion (demographic and clinical characteristics). For the purpose of questionnaire analysis, a total of 23 numerical values corresponding to subscales of the FIS-40, PSQI, HADS, and SF-36 outcome measures were used. For each one, two samples collected at different times were analyzed, as shown in the CONSORT chart. For variables describing demographic and clinical characteristics, such as age, BMI, heart rate, and medication use, a total of four numerical and five dichotomous variables obtained at baseline were analyzed. The normality of the data was evaluated for all numerical variables using the Shapiro–Wilk test, and the statistical value W was calculated using the appropriate formula. For all cases, results were considered significant for an alpha value = 0.05, and the *p*-value was reported with four digits or as indicated if the value was less than 0.001. All statistical analyses were performed using Python package Pingouin (version 0.5.2) for Windows (University of California, Berkeley, CA, USA).

$$W=\frac{{\left(\sum_{i=1}^{n}\;a_{i}\;x_{i}\right)}^{2}}{\sum_{i=1}^{n}\;{\left(x_{i}-\bar{x}\right)}^{2}}$$

#### 2.9.1. Independent Sample Analysis

The normality of independent samples was analyzed using the Welch–Satterthwaite equation, an approximation of the adjusted degrees of freedom, since the sizes were assumed to be unequal. The formula used was as follows (Delacre, Lakens, and Leys, n.d.):

$$v=\frac{{\left(\frac{s_{x}^{2}}{n_{x}}+\frac{s_{y}^{2}}{n_{y}}\right)}^{2}}{\frac{{\left(\frac{s_{x}^{2}}{n_{x}}\right)}^{2}}{\left(n_{x}-1\right)}+\frac{{\left(\frac{s_{y}^{2}}{n_{y}}\right)}^{2}}{\left(n_{y}-1\right)}}$$

The Mann–Whitney *U* test, a nonparametric test of the null hypothesis in which a value chosen at random from a sample is equally likely to be less than or greater than a random value, was analyzed. A brute force version of the formula from Vargha and Delaney, 2000, was used. For all cases, results were considered significant for an alpha value = 0.05, and the *p*-value was reported with four digits or as indicated if the value was less than 0.001.

#### 2.9.2. Paired Data Analysis

To analyze paired samples of questionnaires whose values were collected at different times, the paired sample t-test was used to assess the data normality. If data were non-normal, the Wilcoxon signed-rank test was applied.

#### 2.9.3. Categorical Data Analysis

To evaluate the balance of the samples of the two groups, the Chi-squared test of independence was used consisting of a 2 × 2 contingency table, since the variables were dichotomous and the degrees of freedom were 1 in all cases. For all cases, results were considered significant for an alpha value = 0.05, and the *p*-value was reported with four digits or as indicated if the value was less than 0.001.

## 3. Results

### 3.1. Participants’ Characteristics

The baseline demographic and clinical features of the participants are displayed in Table 1. In this study, there was no statistically significant differences in participants’ demographic and clinical data during the intervention (Table 1). The mean (SD) age of patients was 52.90 (6.47) in the intervention arm and 52.5 (7.48) in the placebo. All participants had a normal BMI with means of 23.58 (3.15) and 22.95 (2.83), for intervention and placebo, respectively. All received medications comprised anticonvulsants, antidepressants, anxiolytics, analgesics, and NSAIDs.

### 3.2. Changes in Fatigue Perception

**Figure 3:**
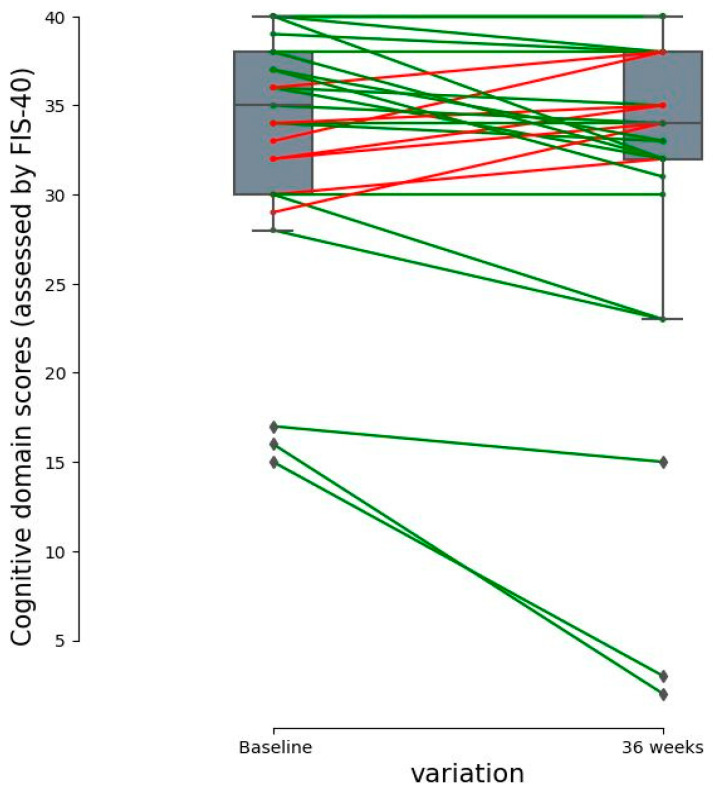
Dot plot of individual paired data of the cognitive fatigue domain (assessed using FIS-40) with significant differences. The differences between the values of the cognitive subscale domain are shown. Each point represents the score of each patient. Bars depict group medians. Lines indicate that cognitive scores belong to the same patient. Green lines depict a decrease in the perceived cognitive fatigue score (better cognitive function), and the red lines indicate an increase in the perceived cognitive fatigue score (poor cognitive function) from the baseline as quantified on the *Y* axis.

Cognitive fatigue (Table 2), as indicated by the change in the cognitive domain, improved significantly from the baseline at the 36-week visit in the intervention group (*p* = 0.0338). The FIS-40 domain scores evolved in parallel between groups over the course of the study. The actual percentage difference was 5.7%, so the improvement effect could be due to the patient-self-reported interindividual variability and/or synergic effects of beta-glucans plus multivitamins among other potential effects (Figure 3).

### 3.3. Changes in Sleep Quality, Anxiety/Depression, and Health-Related Quality of Life

#### 3.3.1. Sleep Quality Assessment

The changes observed in the sleep quality between study groups from the baseline to the final assessment are displayed in Table 3. The intervention with dietary supplements significantly improved daytime dysfunction at the 36-week visit in the active group (*p* = 0.0131 with respect to the baseline) compared to the placebo group. The other PSQI domain scores of the two groups evolved in parallel over the course of the study.

#### 3.3.2. Anxiety and Depression

As shown in Table 4, the anxiety and depression symptoms did not present statistical differences between groups over the course of the study.

#### 3.3.3. Health-Related Quality of Life

As display in Table 5, social role functioning improved significantly at the 36-week visit compared to the baseline in the placebo group (*p* = 0.0131). The SF-36 domain scores evolved in parallel between groups over the course of the study.

### 3.4. Clinical Safety and Intervention Tolerability

No relevant treatment-related adverse events were recorded in the study population. The intervention with dietary supplement containing yeast-derived beta-glucans + vitamin D3 + vitamin B6 + zinc over the period of 9 months was safe and well tolerated by the patients.

## 4. Discussion

Emerging evidence linking alterations in the gut microbiota composition with the clinical symptoms reported by ME/CFS patients suggests a potential therapeutic role for microbiome-targeted interventions, such as prebiotic supplementation. By restoring intestinal homeostasis, this approach may help alleviate sufferers’ health complaints and improve their quality of life. Therefore, to investigate the advantages of the proposed intervention, this clinical trial was designed to evaluate the effect of a food supplement ImmunoVita^®^, containing yeast-derived beta-glucan, combined with vitamin D3, vitamin B6, and zinc, on fatigue, sleep problems, anxiety/depression, and overall quality of life in ME/CFS patients.

In this study, beta-glucan supplements were administered to individuals with ME/CFS over a prolonged time period (36 weeks). A slight reduction in cognitive fatigue symptoms was reported along with an improvement in self-reported HR-QoL in the active arm of the study participants. Another controlled study assessing the effects of beta-glucan supplementation in ME/CFS patients over a 12-week period showed a significant reduction in patients’ self-perceived fatigue and an improvement in their immune function compared to the placebo group [42].

The main findings of this study provide early evidence that administration of beta-glucan may influence certain neurocognitive outcomes related to perceived fatigue in ME/CFS patients after 36 months’ treatment. The outcomes are consistent with the growing body of evidence supporting the presence of a two-way hormonal and neural signaling pathway between the gut microbiome–brain axis and its ability to influence metabolism, behavior, and neurocognitive functions through the production of microbial by-products exerting various local and systemic effects on gut hormones, oxidative stress, and inflammation in ME/CFS [21,43,44,45]. The gut–brain axis is considered to be a key regulator of cognitive function. In the present study, we demonstrated beneficial effects of beta-glucan supplementation on cognitive fatigue in ME/CFS and presented the first evidence of 36 weeks of beta-glucan supplementation improving cognitive impairment, assessed using FIS-40 measures.

Cognitive fatigue became less severe after the 36-week treatment with beta-glucan, as did exhaustion, but the differences with respect to placebo were not significant. The lack of cognitive energy was less severe in the active group compared to the baseline. These results are in line with pre-clinical studies showing that beta-glucan attenuates cognitive impairment via the gut–brain axis in diet-induced obese mice [46,47,48]. Experimental studies using murine models have shown that beta-glucan treatment can exert significant anti-fatigue effects, as demonstrated by the increased exhaustive swimming time in mice during the forced swimming test [30]. These benefits were attributed to the potential beneficial effects of beta-glucan on energy metabolism and oxidative stress, demonstrated in the form of reduced levels of exercise fatigue and injury-related blood biomarkers, including lactate, blood urea nitrogen (BUN), creatinine kinase (CK), alanine transaminase (ALT), and aspartate transaminase (AST), reduced serum glucose, and improvements in the response to exercise-induced oxidative stress [30].

Evidence from human trials has shown that increasing prebiotic fiber intake can influence perceived fatigue. For example, consuming cereals high in wheat fiber for two weeks reduced fatigue compared to the control in healthy adults [49], whereas a 13-week long supplementation with a prebiotic containing inulin and fru-oligosaccharides reduced exhaustion in elderly subjects when compared to the control [50]. In healthy adults, supplementation with oats (1 g), providing 1.9 g of dietary fiber as beta-glucan, significantly decreased the occurrence of exhaustion and fatigue compared with the baseline (*p* < 0.05). Interestingly, individuals who received beta-glucan had less severe headaches and lower perception of cold than controls. In addition, changes in inflammatory markers, C-reactive-protein (CRP), and oxidized-LDL with gastrointestinal symptom severity were associated with the occurrence and severity of several non-GI symptoms [51]. Furthermore, the results of a phase I/II clinical trial conducted in patients with advanced malignancies receiving chemotherapy indicated that beta-(1,3)-(1,6)-D-glucan administered as an adjunctive therapy may improve white blood cells and platelet counts, and may also raise levels of hemoglobin, which in up to 40% of patients reduces the feeling of fatigue compared with the period before the supplementation [52].

Fatigue is associated with a lack of sleep, an association that may be mediated by increased inflammation [53]. To date, the therapeutic effects of beta-glucans in ME/CFS have not been assessed. Beta-glucans are naturally occurring polysaccharides found in the cell walls of fungi, algae, bacteria, and some cereals. It has been suggested that they may have immunomodulatory and anti-inflammatory effects, which could be beneficial for ME/CFS. Some preliminary studies have shown improvements in ME/CFS patients’ perception of fatigue after beta-glucan administration [54].

In relation to sleep disturbances and anxiety/depression, there is limited evidence on the effects of beta-glucan on these symptoms in ME/CFS. Some studies have suggested that beta-glucan may have anxiolytic and antidepressant properties, but further research is required to support these claims and to determine its efficacy in this situation [55]. In a recent study assessing the effects of beta-glucan administration on mental health and HPA axis, reactivity was assessed in ME/CFS, and this approach improved anxiety/depression and HPA axis function [56]. In another study, apparently healthy adults who regularly consume an oats-based supplement containing 1.9 g of dietary fiber like beta-glucan had significantly fewer feelings of anxiety/depression [30].

Another preliminary study found that beta-glucan administration improved mood and vitality among HR-QoL measures in ME/CFS [51]. Overall, while there are some preliminary research studies suggesting possible potential benefits of beta-glucan administration on fatigue, mental health, and quality of life in ME/CFS, the current evidence is limited, and more research is needed to fully evaluate its efficacy and understand the pathophysiological mechanisms after beta-glucan administration in ME/CFS and other chronic post-infectious conditions.

These previous studies suggest that beta-glucans have potential beneficial effects in ME/CFS, including improving quality of life, reducing fatigue, decreasing anxiety/depression, and modulating the immune response. However, research on this topic is still ongoing, and more studies are needed to confirm and extend these findings, and also to determine the different sources, functionality (viscosity, fermentability, solubility, etc.), optimal doses, and duration of beta-glucan treatment that may affect physiological outcomes in ME/CFS.

The current results provide consistent evidence linking increased beta-glucan intake to enhanced cognitive function in ME/CFS. The beneficial effect suggests that the relationship between gut microbiota alteration and cognitive impairment may be causal. In addition to highlighting the adverse impact of western diets on the gut–brain axis, the findings of this study suggest that enhanced consumption of beta-glucan-rich foods is an easily implementable nutritional strategy for attenuating the diet-induced cognitive decline in ME/CFS patients [57].

### Strengths and Limitations

This study has several strengths and some limitations that must be considered. To the best of our knowledge, this trial is the first clinical evaluation of the effects of beta-glucan supplementation with multivitamins (including vitamin D3, vitamin B6, and zinc) involved in energy metabolism and immune function enhancement on ME/CFS patients’ perceptions of their neurocognitive symptoms, fatigue, sleep quality, and anxiety/depression. It should be noted that a cognition improvement found through percentage differences of 5.7% in active arm is more likely to be due to a real treatment effect using beta-glucans plus multivitamins among the participants. In addition, study participants were assessed according to the ME/CFS case criteria based on the 1994 CDC/Fukuda definition, and a potential gender bias was avoided by restricting the study to females.

The main limitation of this study was the relatively small sample size. In addition, these participants were recruited from a single tertiary referral center, so the results cannot be generalized to other ME/CFS populations. It should also be noted that the primary endpoint, the perception of fatigue assessed with the FIS-40 questionnaire, was a self-reported parameter. Finally, the lack of statistical differences between groups may suggest a placebo effect for microcrystalline cellulose and plant capsule.

In future studies, the doses and follow-up timing of the intervention should be pre-established, so as to ensure appropriate monitoring beyond the intervention. In addition, objective physiological and biological parameters should be used to evaluate beneficial effects of beta-glucan in ME/CFS and other post-viral fatigue syndromes.

## 5. Conclusions and Future Directions

These findings provide hypothesis-generating evidence that beta-glucan may be beneficial for several affective and physical states in ME/CFS. For example, its positive effect on cognitive function and gut microbiome could be extended and applied in a disease model of cognitive decline. Since information is lacking on the effects on non-GI symptoms in ME/CFS of dietary fibers in general, and of beta-glucan in particular, these results provide potentially useful data for the design of controlled studies required to confirm these observations.

This study provides the first evidence that beta-glucan administration improves indices of cognition function with major beneficial effects all along the gut microbiota–immune–brain axis. Our data suggest that the consumption of 1 g of beta-glucan is an easily implementable nutritional strategy for alleviating the detrimental features of gut–brain dysregulation and for preventing cognitive decline in ME/CFS.

## Figures and Tables

**Table 1:** Baseline demographic and clinical features for each arm of the study participants.

Variables	Active (n = 29)	Placebo (n = 22)	*p*-Values
Age (years)	52.90 ± 6.47	52.50 ± 7.48	0.90
BMI (kg/m^2^)	23.58 ± 3.15	22.95 ± 2.83	0.39
Heart rate (bpm)	75.27 ± 11.69	74.50 ± 7.71	1.00
Concomitant drugs			
Anticonvulsants	9	6	0.98
Antidepressants	24	16	0.60
Anxiolytics	19	14	0.87
Analgesics	21	13	0.48
NSAIDs	6	5	0.86

Data are expressed as means ± standard deviation (SD) for continuous variables. In both cases, the non-parametric Mann–Whitney U test was used because the samples were not normally distributed. No significant differences were observed in either case, confirming that both samples were derived from the same population. The Chi-squared test of independence was performed with one degree of freedom, and no significant differences in the proportions were observed, so no variable was considered to exert a different influence depending on the group. Abbreviations: BMI, body mass index; NSAIDs, non-steroidal anti-inflammatory drugs.

**Table 2:** Changes in the fatigue severity assessed with the FIS-40 score from the baseline to the final assessment (at week 36) in the study participants.

FIS-40 Domains	Baseline	36-Weeks	*p*-Values ^1^
Active arm (n = 29)			
Cognitive	33.31 ± 7.03	31.45 ± 9.76	0.0338 *
Psychosocial	61.79 ± 14.59	60.45 ± 16.92	0.3681
Physical functioning	34.52 ± 5.20	33.28 ± 6.78	0.0725
Total FIS-40 score	129.62 ± 25.37	125.17 ± 31.43	0.1070
Placebo (n = 22)			
Cognitive	33.82 ± 5.42	32.73 ± 5.76	0.3725
Psychosocial	64.14 ± 11.79	59.45 ± 12.92	0.1398
Physical functioning	33.73 ± 5.90	32.32 ± 6.95	0.1313
Total FIS-40 score	131.68 ± 21.16	124.50 ± 23.83	0.1375

Data are expressed as means ± SD. ^1^ The statistical significance of the paired data is analyzed. Statistical significance was set at * *p* < 0.05. The cognitive domain in the active arm presented a slight difference (5.7% from the baseline). Depending on the normality of the data, the test of independence of parametric (*t*-test) or non-parametric (Wilcoxon) paired data was used. There were no significant differences between the groups at baseline. Intervention group was compared with the baseline. Abbreviations: FIS-40, 40-item fatigue impact scale. Lower scores indicate an improvement in fatigue perception. ^1^ *p*-value for intergroup analysis.

**Table 3:** Changes in sleep quality assessed using the PSQI questionnaire from the baseline to the final assessment (week 36).

PSQI Domains	Baseline	36 Weeks	*p*-Values ^1^
Active arm (n = 29)			
Subjective sleep quality	2.14 ± 0.88	2.17 ± 0.89	0.7389
Sleep latency	2.07 ± 1.03	2.14 ± 1.16	0.5271
Sleep duration	1.69 ± 1.00	1.62 ± 0.98	0.4795
Habitual sleep efficiency	1.93 ± 1.33	1.83 ± 1.20	0.5728
Sleep disturbances	2.10 ± 0.72	2.21 ± 0.73	0.1797
Use of sleeping medication	2.07 ± 1.31	2.00 ± 1.39	0.6256
Daytime dysfunction *	2.31 ± 0.89	2.28 ± 0.75	0.7963
Overall PSQI score	14.31 ± 4.91	14.24 ± 4.93	0.8845
Placebo (n = 22)			
Subjective sleep quality	2.05 ± 0.84	1.68 ± 0.99	0.0588
Sleep latency	2.32 ± 0.78	2.18 ± 0.85	0.3173
Sleep duration	1.41 ± 1.05	1.36 ± 1.09	0.7630
Habitual sleep efficiency	1.77 ± 1.31	1.59 ± 1.40	0.2575
Sleep disturbances	2.09 ± 0.53	1.95 ± 0.58	0.2568
Use of sleeping medication	1.95 ± 1.36	2.14 ± 1.17	0.3795
Daytime dysfunction *	1.59 ± 0.73	1.73 ± 0.83	0.4386
Overall PSQI score	13.18 ± 3.85	12.64 ± 4.70	0.4567

Data are expressed as means ± SD. ^1^ The statistical significance of the paired data is analyzed. Depending on the normality of the samples, parametric (*t*-test) or non-parametric (Wilcoxon) tests for paired data were used. Baseline data showed no significant differences between the groups, indicating that there was no bias at the baseline between the two groups, (*) except in daytime dysfunction, in which significant differences were found using the Mann–Whitney U test for independence with a *p*-value of 0.0015; therefore, this variable should not be considered.

**Table 4:** Changes in the anxiety/depression symptoms assessed through the HADS questionnaire from the baseline to the final assessment (at week 36).

FIS-40 Domains	Baseline	36-Weeks	*p*-Values ^1^
Active arm (n = 29)			
Anxiety	12.86 ± 5.05	12.76 ± 4.70	0.7940
Depression	11.86 ± 5.11	11.86 ± 5.59	0.5354
Total HADS	24.72 ± 9.60	24.62 ± 9.67	0.6716
Placebo (n = 22)			
Anxiety	11.82 ± 4.55	11.00 ± 4.65	0.1821
Depression	11.59 ± 4.07	10.68 ± 3.98	0.1218
Total HADS	23.41 ± 8.19	21.68 ± 8.20	0.1890

Data are expressed as means ± SD. ^1^ The statistical significance of the paired data is analyzed. Depending on the normality of the samples, parametric (*t*-test) or non-parametric (Wilcoxon) tests for paired data were used. Baseline data showed no significant differences between the groups, indicating that there was no bias at the baseline between the two groups.

**Table 5:** Changes in the health-related quality of life evaluated using the SF-36 questionnaire from the baseline to the final assessment (at week 36).

SF-36 Domains	Baseline	36-Weeks	*p*-Values ^1^
Active arm (n = 29)			
Physical functioning	38.45 ± 20.49	40.17 ± 22.50	0.8178
Physical role functioning	6.03 ± 16.66	10.34 ± 20.61	0.2020
Bodily pain	15.93 ± 15.58	20.90 ± 18.68	0.1468
General health perception	22.76 ± 14.18	21.90 ± 13.19	0.5683
Vitality	12.24 ± 13.53	16.55 ± 15.93	0.0526
Social role functioning	32.33 ± 26.63	36.64 ± 25.65	0.3344
Emotional role functioning	34.48 ± 46.70	28.74 ± 43.39	0.1695
Mental health status	38.21 ± 21.18	42.07 ± 22.69	0.1393
Placebo (n = 22)			
Physical functioning	39.32 ± 21.17	38.18 ± 20.21	0.9243
Physical role functioning	1.14 ± 5.33	4.55 ± 16.61	0.3287
Bodily pain	20.14 ± 19.22	23.73 ± 15.33	0.1089
General health perception	25.32 ± 14.49	27.36 ± 17.57	0.3225
Vitality	17.27 ± 16.24	15.23 ± 12.77	0.5847
Social role functioning	30.68 ± 20.68	42.05 ± 22.34	0.0131 *
Emotional role functioning	33.33 ± 44.84	37.88 ± 45.19	0.4797
Mental health status	38.73 ± 23.11	44.73 ± 20.01	0.1918

Data are expressed as means ± SD. ^1^ The statistical significance of the paired data is analyzed. (*) Social role functioning in the placebo group presented a significant difference. Depending on the normality of the samples, parametric (*t*-test) or non-parametric (Wilcoxon) tests for paired data were used. Baseline data were analyzed between the two groups, indicating that there was no bias at the baseline between the two groups.

## Data Availability

All relevant data analyzed during the current trial are included in the article. Access to raw datasets may be shared upon reasonable request to the corresponding author.
